# Impact of Reimbursement on the Utilisation of Gene Expression Profiles and Chemotherapy Decision‐Making in Dutch Breast Cancer Patients: A Population‐Based Study

**DOI:** 10.1002/ijc.70571

**Published:** 2026-06-04

**Authors:** Joyce Meijer, Nienke Hermanns, Rhodé Bijlsma, Hedwig M. Blommestein, Desiree H. J. G. van den Bongard, Thijs van Dalen, Paul J. van Diest, Caroline A. Drukker, Cristina Guerrero Paez, Aafke H. Honkoop, Agnes Jager, Linetta B. Koppert, Sabine C. Linn, Marissa C. van Maaren, Ester J. Siemerink, Laura J. van ’t, José H. Volders, Marie Jeanne Vrancken Peeters, Sabine Siesling

**Affiliations:** ^1^ Department of Research and Development Netherlands Comprehensive Cancer Organisation (IKNL) Utrecht the Netherlands; ^2^ Department of Health Technology and Services Research Technical Medical Centre, University of Twente Enschede the Netherlands; ^3^ Division of Imaging and Oncology University Medical Center Utrecht, Utrecht University Utrecht the Netherlands; ^4^ Erasmus School of Health Policy & Management Rotterdam the Netherlands; ^5^ Department of Radiation Oncology Amsterdam UMC Amsterdam the Netherlands; ^6^ Cancer Center Amsterdam, Cancer Treatment and Quality of Life/Cancer Biology and Immunology Amsterdam the Netherlands; ^7^ Department of Surgery Erasmus Medical Centre Rotterdam the Netherlands; ^8^ Department of Pathology University Medical Center Utrecht Utrecht the Netherlands; ^9^ Department of Surgical Oncology Netherlands Cancer Institute Amsterdam the Netherlands; ^10^ Dutch Breast Cancer Society (BVN) Utrecht the Netherlands; ^11^ Department of Medical Oncology Isala Clinics Zwolle the Netherlands; ^12^ Department of Medical Oncology Erasmus Medical Center Cancer Institute Rotterdam the Netherlands; ^13^ Department of Medical Oncology Netherlands Cancer Institute Amsterdam the Netherlands; ^14^ Department of Pathology University Medical Centre Utrecht Utrecht the Netherlands; ^15^ Department of Medical Oncology Ziekenhuisgroep Twente Hengelo the Netherlands; ^16^ University of California San Francisco San Francisco California USA; ^17^ Department of Surgery Diakonessenhuis Utrecht the Netherlands; ^18^ Department of Surgery Amsterdam University Medical Center Amsterdam the Netherlands

**Keywords:** breast cancer, chemotherapy, gene expression profile, population‐based, reimbursement

## Abstract

This study examined gene expression profile (GEP) use in relation to reimbursement policies and chemotherapy administration, both for patients with and without GEP indication. Women aged ≥ 18–69 years and diagnosed with early‐stage breast cancer between January 2011 and June 2024 were selected from the Netherlands Cancer Registry. GEP use in relation to reimbursement policies and GEP result in relation to chemotherapy administration was analysed using flowcharts and a trend line. For patients with GEP indication, logistic regression analyses were conducted to determine if the reimbursement periods influenced the GEP use, and GEP use was analysed per region in the Netherlands and incidence year using trend lines. In total, 138,765 patients were included. Among 17,525 patients with GEP indication, proportions of patients who received a GEP were higher during retrospective reimbursement (25%) and publicly known reimbursement (37%) compared with no reimbursement (9%) and differed across regions in the Netherlands. Proportions of patients who did not receive chemotherapy were higher for patients who had a low‐risk MammaPrint (93%) or Oncotype DX (97%) result compared with patients who had a high‐risk MammaPrint (17%) or Oncotype DX (15%) result. Among 13,003 patients with GEP indication who did not receive GEP, 52% did receive chemotherapy. Approximately one‐third of patients who met GEP criteria and were eligible for publicly known reimbursement received a GEP. Appropriate follow‐up steps and further research seem required to analyse reasons for not ordering a GEP in times of reimbursement, since GEP results help chemotherapy decision‐making.

## What’s New?

To avoid unnecessary treatment in breast cancer, several gene expression profiling tests have been developed to help clinicians identify patients who may not benefit from chemotherapy. This study examined the effect of reimbursement availability on the implementation of gene expression profiles using population‐based data from the Netherlands Cancer Registry. Reimbursement availability was somewhat efficacious for the implementation of profiling tests, although only one‐third of patients eligible for publicly known reimbursement received a test. Chemotherapy use was reduced for low‐risk gene expression profiles. The reasons for not ordering a test in times of reimbursement remain unclear, since profiling supports chemotherapy decision‐making.

## Graphical abstract

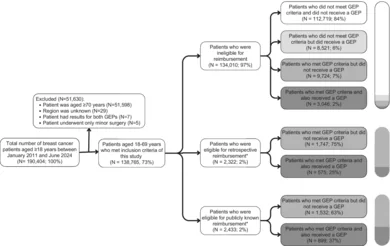

**Abbreviations**
CBS: Statistics Netherlands
ER: Oestrogen receptor
GEP: gene expression profile
HER2: human epidermal growth factor receptor 2
HR: hormone receptor
IQR: interquartile range
NCR: Netherlands Cancer Registry
OR: odds ratio
PR: progesterone receptor
SEP: socioeconomic position
ZINL: Dutch National Care Institute

## 1 Introduction

Breast cancer is a commonly occurring type of cancer worldwide and particularly affects women in Western nations such as the Netherlands [1]. Representing 25% of all cancer diagnoses, breast cancer is the most prevalent cancer type among Dutch women, especially those aged 50 years and older [2, 3]. Over the years, there has been improvement in breast cancer prognosis, for example, due to timely diagnoses (e.g., supported by the national screening programme), improved imaging and more effective treatments, particularly systemic therapy [2]. Unfortunately, treatments that have improved the prognosis cause side effects, put pressure on healthcare resources and community engagement, for instance, when a patient is no longer able to work and requires informal care. This may be prevented by making treatments more personalised, thereby optimising effectiveness, avoiding overtreatment, avoiding side effects and reducing costs.

To avoid unnecessary treatment with chemotherapy, several gene expression profiles (GEPs) have been developed. MammaPrint, a 70‐gene test, and Oncotype DX, a 21‐gene test, are two GEPs that are used in the Netherlands and both offer insights into the biological behaviour of breast tumours and their potential to cause distant metastasis in the first 5 years after diagnosis [4, 5]. These GEPs assist clinicians to identify patients who have an indication for chemotherapy according to the guideline, but who may not benefit from chemotherapy [6]. Consequently, these GEPs offer a more personalised treatment approach by sparing patients from potentially harmful short‐ and long‐term side effects of chemotherapy in case of a low risk GEP result.

**FIGURE 1:**
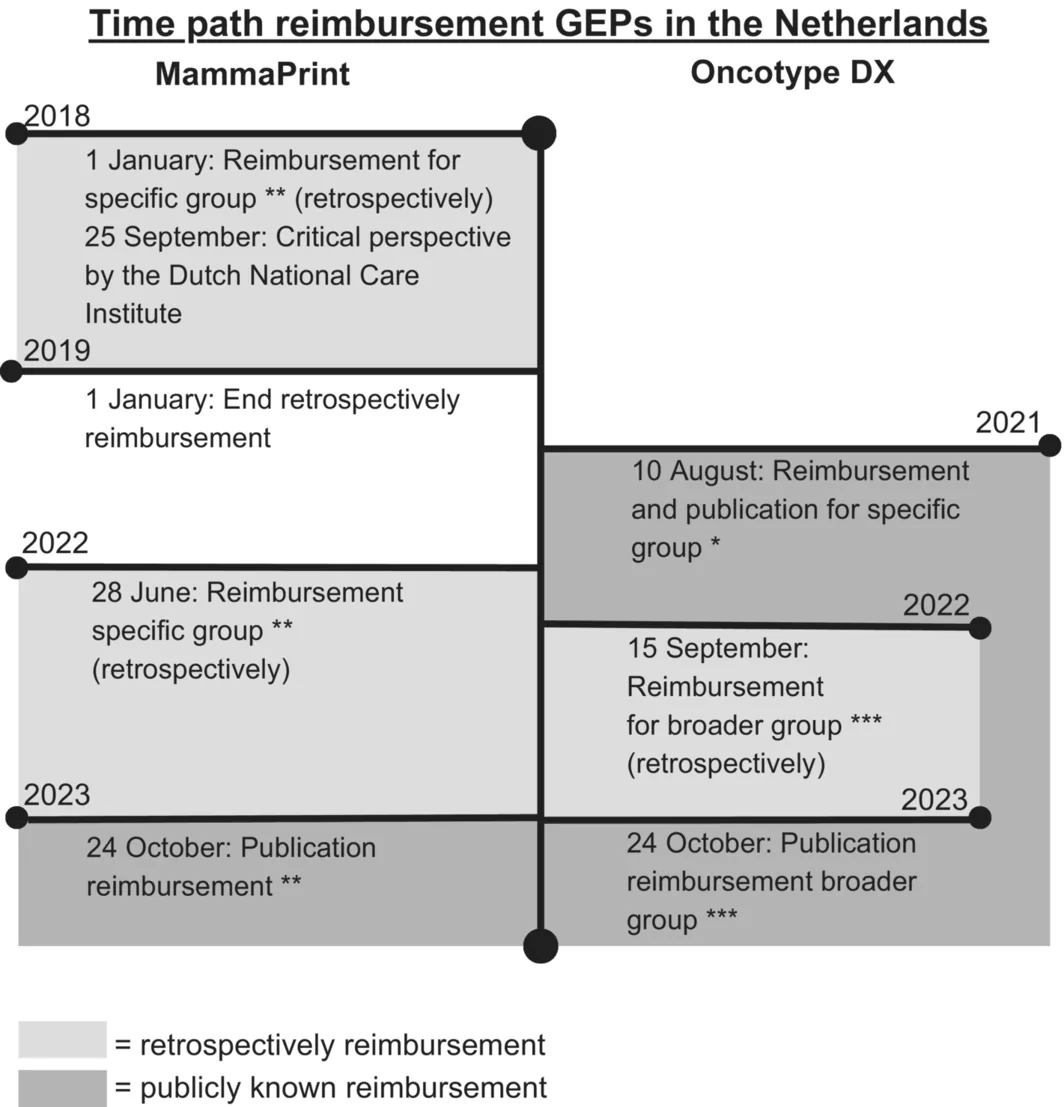
Time path for reimbursement MammaPrint and Oncotype DX in the Netherlands according to the Dutch National Healthcare Institute [11, 12, 13, 14]. *Eligibility criteria: Women > 50 years with a HR+, HER2‐, N0 breast tumour and: A grade 1 and tumour size between 3.1– and 5.0 cm, a grade 2 and tumour size between 2.1 and –5.0 cm, or a grade 3 and tumour size between 1.1 and 2.0 cm. **Eligibility criteria: Women > 50 years with a HR+, HER2‐, N0 breast tumour and: A grade 1 and tumour size between 3.1 and 5.0 cm, a grade 2 and tumour size between 2.1 and 5.0 cm, or a grade 3 and tumour size between 1.1 and 2.0 cm. Additionally for women > 50 years with a HR+, HER2‐, N1 (1–3 axillary lymph node metastases) breast tumour and: A grade 1 and tumour size between 2.0 and 5.0 cm or a grade 2 and tumour size ≤ 5.0 cm. ***Eligibility criteria: Also eligible for women > 50 years with a HR+/HER2‐, N1 (1–3 axillary lymph node metastases) breast tumour and: A grade 1 and tumour size between 2.0 and 5.0 cm or a grade 2 and tumour size ≤ 5.0 cm. GEP, gene expression profile test.

The incorporation of GEPs into clinical practice has the potential to enhance patient outcomes by maintaining quality of life while minimising unnecessary treatments with chemotherapy and limiting healthcare costs since the use of GEPs is proven to be cost‐effective [7, 8, 9, 10]. Adoption of technologies, including GEPs, is influenced by several factors such as reimbursement policies. In the Netherlands, the reimbursement of diagnostics and treatments is subject to approval by the Dutch National Care Institute (ZINL). Currently, a reimbursement is levied for GEPs in the Netherlands, though this has not always been the case (Figure 1) [11, 12, 13]. This shift in reimbursement policies for GEP in the Netherlands, as well as the subsequent chemotherapy decisions, have not been studied yet and therefore motivated this study, which aimed to investigate (1) the utilisation of GEP in the context of reimbursement policies, for both patients with and without an indication for a GEP and (2) GEP use in relation to chemotherapy administration, both among women diagnosed with primary invasive breast cancer without distant metastasis who underwent treatment with curative intent between January 2011 (when GEP use and GEP results were first registered) and June 2024 in the Netherlands.

## 2 Methods

### 2.1 Data Collection

The Netherlands Cancer Registry (NCR) includes all newly diagnosed malignancies notified through the Dutch Nationwide Pathology Databank (Palga) since 1989. Data were selected from the NCR and derived from 76 hospitals in the Netherlands, consisting of 41 general, 27 top‐clinical and eight academic hospitals. The data were collected by trained data managers and obtained directly from the patient files. Since 2011, data regarding GEP use and GEP results have been registered in the NCR.

### 2.2 Study Population

**FIGURE 2:**
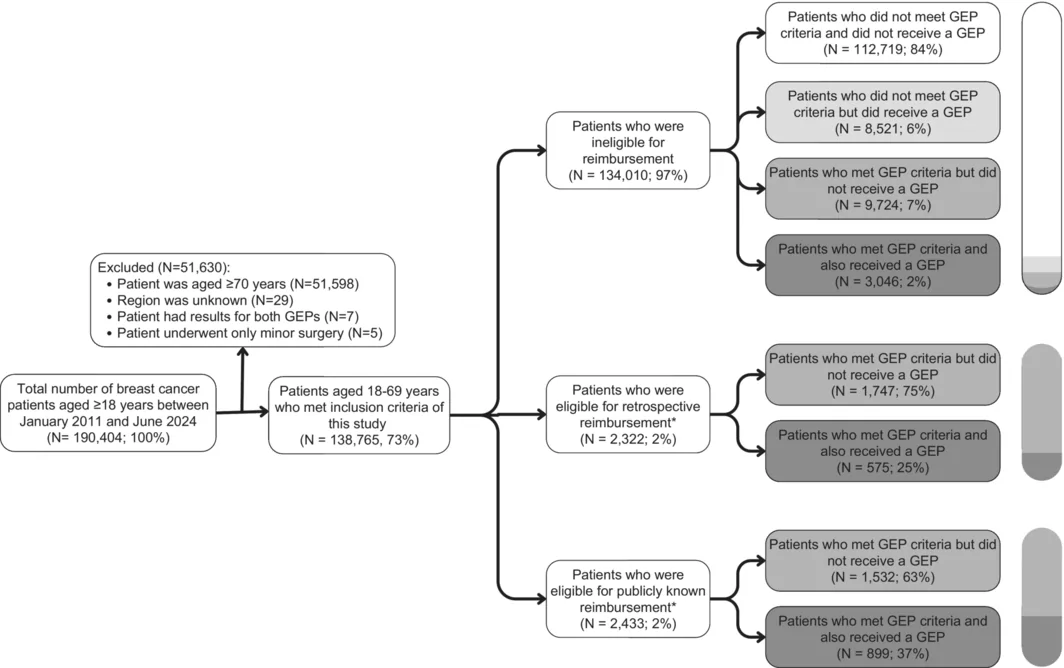
Number of included patients grouped according to reimbursement criteria, gene expression profile (GEP) criteria and GEP use. Eligibility for reimbursement was based on all information provided in Figure 1. Eligibility for GEP criteria was based on: Women, older than 50 years, HR+, HER2‐ and either N0 status, grade 1 tumour between 3.1 and 5.0 cm or grade 2 between 2.1 and 5.0 cm or a grade 3 between 1.1 and 2.0 cm, or N1 status and 1–3 axillary lymph node metastases, a grade 1 tumour between 2.1 and 5.0 cm or a grade 2 tumour ≤ 5.0 cm. Hereby, changes in criteria for GEP as well as reimbursement over time were taken into account. The year 2024 comprised solely the months January to June. *Patients who were eligible for retrospective or publicly known reimbursement all met GEP criteria.

Women aged 18–69 years and diagnosed with primary invasive breast cancer without distant metastasis (M0) between January 2011 and June 2024 in the Netherlands were selected for this study. Patients who received both MammaPrint and Oncotype DX and therefore could not be placed in one group, patients who underwent minor surgery (e.g., local tumour resection or lymph node dissection), and patients whose region of treatment was unknown were excluded from the analysis (Figure 2).

Patients aged ≥ 70 years were ineligible for chemotherapy in the Netherlands, unless they had no comorbidities with a good performance status [14]. As data concerning comorbidities and performance status were unavailable in the NCR for breast cancer patients, all patients aged ≥ 70 years were assumed to be ineligible for chemotherapy and therefore not included in this study.

### 2.3 Definitions

For all patients, age at diagnosis was grouped into age categories ≤ 50, 51–59, 60–69. The TNM classification (8th edition) was used for staging the tumour [15]. If the pN status was missing, the cN status was used instead. The Modified Bloom‐Richardson Grading System was used for grading: grade 1, 2 or 3 [16]. The tumour size classification aligned with the reimbursement criteria for both GEPs: < 1.1 cm, 1.1–2.0 cm, 2.1–3.0 cm, 3.1–5.0 cm and > 5.0 cm. Oestrogen receptor (ER), progesterone receptor (PR), and human epidermal growth factor receptor 2 (HER2) were classified as positive or negative. Hormone receptor (HR) positivity was defined as PR and/or ER had positivity in ≥ 10% of invasive tumour cells. HER2 positivity was defined as a positive immunohistochemistry (3+, at least 10% of cells with membrane staining of strong intensity). In case of a 2 positive immunohistochemistry score (at least 10% of cells stained with moderate intensity), an amplification test was performed that overruled the results of the immunohistochemistry. Socioeconomic position (SEP) was determined based on the six‐digit postal code of each patient, using median household income as a proxy for SEP. Statistics Netherlands (CBS) provided household income data at the six‐digit postal code level [17]. To investigate whether there were regional differences regarding GEP use, hospitals where patients received their treatment for breast cancer were categorized into seven different regions in the Netherlands (Mid, Northeast, Northwest, East, West, Southeast and Southwest), and into three different types of hospital (general, top‐clinical and academic). MammaPrint results were classified as high risk or low risk. The low, intermediate, and high Oncotype DX results were based on the original recurrence score cutoffs of < 18, 18–30 and ≥ 31, respectively [18, 19]. Date of diagnosis was used if the date of the test was not documented. Dates were presented in month and year.

The following criteria were used to determine patients' eligibility for MammaPrint or Oncotype DX [4, 5, 11, 12, 13, 20]: women, older than 50 years, HR+, HER2‐ and either N0 status, grade 1 tumour between 3.1 and 5.0 cm or grade 2 between 2.1 and 5.0 cm or a grade 3 between 1.1 and 2.0 cm, or N1 status and 1–3 axillary lymph node metastases, a grade 1 tumour between 2.1 and 5.0 cm or a grade 2 tumour ≤ 5.0 cm.

Based on these criteria combined with the timeline of reimbursement (Figure 1), patients were divided in three different groups: (1) patients who were ineligible for reimbursement, (2) patients who were eligible for retrospective reimbursement and (3) patients who were eligible for publicly known reimbursement. Hereby, changes in criteria over time were also taken into account.

Retrospective reimbursement was based on periods when ZINL instituted retrospective reimbursement (Figure 1). For example, for MammaPrint, it was decided on 24 October 2023 that retrospective reimbursement was instituted from 28 June 2022. So, the period from 28 June 2022 to 24 October 2023 is a retrospective reimbursed period. Hence, the period from 24 October 2023 onwards was then seen as publicly known reimbursement.

### 2.4 Statistical Analysis

The main analyses of this study were performed for women aged 18–69 years and diagnosed with primary invasive breast cancer without distant metastasis (M0) between January 2011 and June 2024 in the Netherlands.

Descriptive statistics were performed with regard to patient and tumour characteristics and tested by Chi‐squared tests. A flowchart was created to show GEP use during different reimbursement circumstances, for both patients who did meet or did not meet GEP criteria. Furthermore, trends over time were shown by calculating the proportion of patients who received a MammaPrint, Oncotype DX, or no GEP per incidence year. In addition, the proportion of patients who received a MammaPrint, an Oncotype DX or no GEP were compared per region and incidence year to assess whether hospitals in different regions switched from MammaPrint to Oncotype DX related to reimbursement. Another flowchart was created to show the GEP result in relation to chemotherapy decisions. Lastly, logistic regression analyses were conducted exclusively on patients who met GEP criteria and conducted separately for MammaPrint and Oncotype DX while adjusting for age, tumour size, lymph node involvement, tumour grade, SEP and region in the Netherlands to determine if the reimbursement periods influenced the GEP use. First, a univariable analysis was conducted (*p* < 0.1), followed by a multivariable analysis (*p* < 0.05).

Stata version 17.0 software was used for all analyses [21].

## 3 Results

Patient and tumour characteristics of the 138,765 included patients are provided in Table 1.

**TABLE 1:** Patient‐ and tumour‐related characteristics of patients aged 18–69 years and diagnosed with primary invasive breast cancer without distant metastasis between January 2011 and June 2024 treated with curative intent in The Netherlands (*N* = 138.765).

	Total (*N* = 138,765)	1. No GEP (*N* = 125,722)	2. MammaPrint (*N* = 11,427)	3. Oncotype DX (*N* = 1,616)	*p* 1 versus 2	*p* 1 versus 3	*p* 2 versus 3
Age (Median, IQR) (*N*, %)	56 (49–63)	56 (49–63)	55 (49–62)	58 (51–63)			
≤ 50	44,697 (32)	40,797 (32)	3,550 (31)	350 (22)	< 0.001	< 0.001	< 0.001
51–59	41,092 (30)	36,649 (29)	3,878 (34)	565 (35)			
60–69	52,976 (38)	48,276 (38)	3,999 (35)	701 (43)			
Stage of primary tumour (*N*, %)							
1	71,014 (51)	64,790 (52)	5,658 (50)	566 (35)	< 0.001	< 0.001	< 0.001
2	52,934 (38)	46,356 (37)	5,547 (49)	1,031 (64)			
3	14,817 (11)	14,576 (12)	222 (2)	19 (1)			
Tumour grade (*N*, %)							
1	30,687 (24)	28,942 (25)	1,578 (14)	167 (10)	< 0.001	< 0.001	< 0.001
2	64,156 (50)	55,444 (48)	7,621 (68)	1,091 (68)			
3	34,678 (27)	32,265 (28)	2,067 (18)	346 (22)			
4	17 (0)	17 (0)	0 (0)	0 (0)			
Unknown	9,227	9,054	161	12			
Positive lymph nodes (*N*, %)							
0	94,445 (70)	85,953 (71)	7,386 (66)	1,106 (70)	< 0.001	< 0.001	< 0.001
1–3	33,179 (25)	28,991 (24)	3,709 (33)	479 (30)			
4–9	4,381 (3)	4,285 (4)	93 (1)	3 (0)			
≥ 10	2,212 (2)	2,179 (2)	32 (0)	1 (0)			
Unknown	4,548	4,314	207	27			
Tumour size (cm) (*N*, %)							
≤ 1.0	45,316 (33)	44,268 (35)	965 (8)	83 (5)	< 0.001	< 0.001	< 0.001
1.1–2.0	53,035 (38)	45,789 (36)	6,542 (57)	704 (44)			
2.1–3.0	20,692 (15)	17,183 (14)	2,936 (26)	573 (35)			
3.1–5.0	8,304 (6)	7,379 (6)	727 (6)	198 (12)			
> 5.0	11,418 (8)	11,103 (9)	257 (2)	58 (4)			
Region in the Netherlands (*N*, %)							
Mid	12,681 (9)	11,421 (9)	1,176 (10)	84 (5)	< 0.001	< 0.001	< 0.001
Northeast	18,328 (13)	16,346 (13)	1,830 (16)	152 (9)			
Northwest	28,980 (21)	26,125 (21)	2,680 (23)	175 (11)			
East	22,470 (16)	20,480 (16)	1,814 (16)	176 (11)			
West	13,304 (10)	12,233 (10)	993 (9)	78 (5)			
Southeast	15,254 (11)	14,100 (11)	1,024 (9)	130 (8)			
Southwest	27,748 (20)	25,017 (20)	1,910 (17)	821 (51)			
Type of hospital (*N*, %)							
General	52,423 (38)	46,971 (37)	4,601 (40)	851 (53)	< 0.001	< 0.001	< 0.001
Top‐clinical	72,324 (52)	65,294 (52)	6,301 (55)	729 (45)			
Academic	14,018 (10)	13,457 (11)	525 (5)	36 (2)			
SEP (*N*, %)							
Low	32,237 (24)	29,523 (24)	2,340 (21)	374 (23)	< 0.001	< 0.001	0.011
Intermediate	48,793 (36)	44,291 (36)	3,989 (35)	513 (32)			
High	55,863 (41)	50,201 (40)	4,951 (44)	711 (44)			
Unknown	1,872	1,707	147	18			
Reimbursement policy^a^ (*N*, %)							
No reimbursement	134,010 (97)	122,443 (97)	10,555 (92)	1,012 (63)	< 0.001	< 0.001	< 0.001
Retrospective reimbursement	2,322 (2)	1,747 (1)	481 (4)	94 (6)			
Publicly known reimbursement	2,433 (2)	1,532 (1)	391 (3)	510 (32)			
Chemotherapy (*N*, %)							
No	69,904 (50)	61,646 (49)	7,026 (61)	1,232 (76)	< 0.001	< 0.001	< 0.001
Yes	68,861 (50)	64,076 (51)	4,401 (39)	384 (24)			

*Note:* The year 2024 comprised solely the months January to June. The *p* value was calculated on known values only, using the Chi‐squared test.

Abbreviations: GEP, gene expression profiles; IQR, interquartile range; SEP, socioeconomic position.

^a^ The eligibility criteria for reimbursement were defined in the time path shown in Figure 1.

The median age at diagnosis was 56 years (interquartile range (IQR): 49–63 years). Tumours were mainly stage 1 (51%) or 2 (38%), grade 2 (50%), size ≤ 1.0 cm (33%) or size 1.1–2.0 cm (38%). 70% had no lymph node involvement.

The proportions of patients aged ≤ 50 years or with a stage 1 tumour were higher in patients who received MammaPrint compared with patients who received Oncotype DX, namely 31% and 50% versus 22% and 35%, respectively. Additionally, 92% of patients who received a MammaPrint was not eligible for reimbursement, compared to 63% for patients who received an Oncotype DX. Finally, the proportion of patients who did not receive chemotherapy was higher in patients who received an Oncotype DX (76%) compared with patients who received a MammaPrint (61%).

### 3.1 GEP Use During Different Reimbursement Circumstances

First of all, 134,010 patients were ineligible for reimbursement, 2322 patients were eligible for retrospective reimbursement, and 2433 patients were eligible for publicly known reimbursement (Figure 2). GEP use was 9% before reimbursement, 25% during retrospective reimbursement and 37% during publicly known reimbursement. Therefore, GEP use increased by 16% after the availability of retrospective reimbursement, and 6% more after publicly known reimbursement. Moreover, of the 134,010 patients who were ineligible for reimbursement, 6% did not meet GEP criteria but did receive a GEP anyway, 7% met GEP criteria but did not receive a GEP and 2% met GEP criteria and did receive a GEP.

**FIGURE 3:**
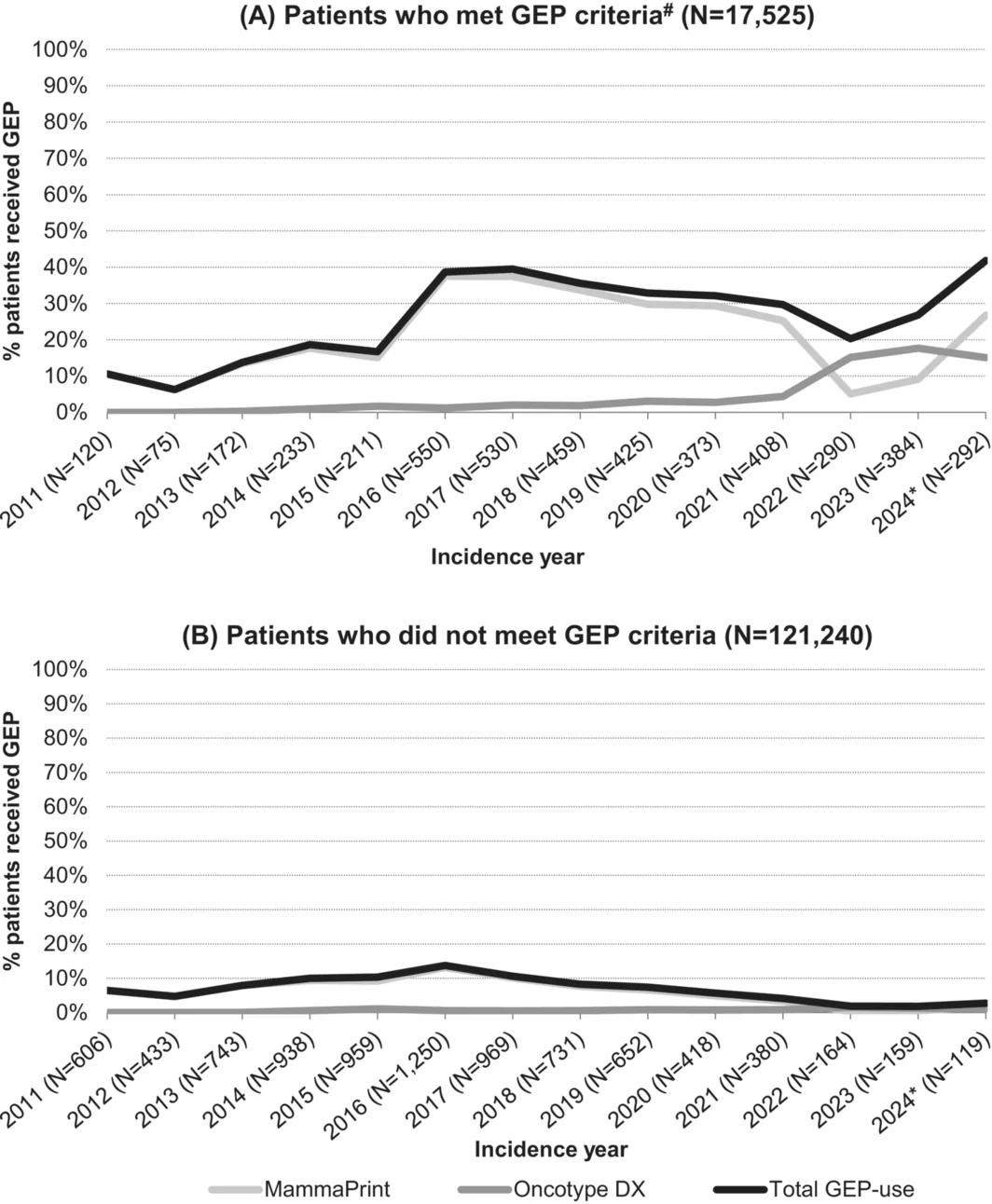
Proportion of patients aged 18–69 years who received a gene expression profile (GEP) test per incidence year for patients diagnosed with primary invasive breast cancer without distant metastasis between January 2011 and June 2024 treated with curative intent in the Netherlands (*N* = 138,765), separately for (A) patients who met GEP criteria and (B) patients who did not meet GEP criteria. N represents the number of patients who received a GEP. ^#^Eligibility for GEP criteria was based on: Women, older than 50 years, HR+, HER2‐ and either N0 status, grade 1 tumour between 3.1 and 5.0 cm or grade 2 between 2.1 and 5.0 cm or a grade 3 between 1.1 and 2.0 cm, or N1 status and 1–3 axillary lymph node metastases, a grade 1 tumour between 2.1 and 5.0 cm or a grade 2 tumour ≤ 5.0 cm. Hereby, changes in criteria for GEP as well as reimbursement over time were taken into account (Figure 1). *The year 2024 comprised solely the months January to June.

For patients who met GEP criteria, there was an increase in the use of MammaPrint between 2015 and 2016, from 15% to 38%, whereafter it declined. The largest decrease was seen from 2021 to 2022, from 25% to 5%. At the same time, there was an increase in the use of Oncotype DX, with the largest increase between 2021 and 2022, from 4% to 15%. In 2024, there was once more an increase in the percentage of patients who received a MammaPrint (Figure 3A).

For patients who did not meet GEP criteria, mostly MammaPrint was performed when a GEP was performed. The same trends were observed as for patients who met GEP criteria, albeit to a lesser extent (Figure 3B).

Patients who met GEP criteria were less likely to receive a MammaPrint while there was retrospective or publicly known reimbursement (odds ratio [4]_retrospect_: 0.97, OR_publicly known_: 0.57, respectively) in comparison with the period during which no reimbursement was received (Table 2). Furthermore, patients who met GEP criteria were more likely to receive an Oncotype DX while there was retrospective or publicly known reimbursement (OR_retrospect_: 2.72, OR_publicly known_: 16.90, respectively) in comparison with the period during which no reimbursement was received.

**TABLE 2:** The influence of reimbursement on gene expression profile (GEP) use of patients aged 18–69 years and diagnosed with primary invasive breast cancer without distant metastasis between January 2011 and June 2024 treated with curative intent in The Netherlands who did meet GEP criteria^a^ (*N* = 17.525).

	Odds ratio (95% CI)	*p*
MammaPrint^b^		
Not eligible for reimbursement	1.00 (reference)	
Eligible for retrospective reimbursement	0.97 (0.87–1.09)	0.638
Eligible for publicly known reimbursement	0.57 (0.51–0.65)	< 0.001
Oncotype DX^b^		
Not eligible for reimbursement	1.00 (reference)	
Eligible for retrospective reimbursement	2.72 (2.11–3.50)	< 0.001
Eligible for publicly known reimbursement	16.90 (14.08–20.29)	< 0.001

*Note:* Multivariable analyses for MammaPrint were adjusted for age, tumour size, lymph node involvement, tumour grade, socioeconomic position and region in the Netherlands. For Oncotype DX, multivariable analyses were adjusted for tumour size, lymph node involvement, tumour grade and region in the Netherlands. The year 2024 comprised solely the months January to June.

Abbreviations: CI, confidence interval; GEP, gene expression profile.

^a^ Eligibility for GEP criteria was based on: women, older than 50 years, HR+, HER2‐ and either N0 status, grade 1 tumour between 3.1 and 5.0 cm or grade 2 between 2.1 and5.0 cm or a grade 3 between 1.1 and 2.0 cm, or N1 status and 1–3 axillary lymph node metastases, a grade 1 tumour between 2.1 and 5.0 cm or a grade 2 tumour ≤ 5.0 cm. Hereby, changes in criteria for GEP as well as reimbursement over time were taken into account.

^b^ The eligibility criteria for reimbursement were defined in the time path shown in Figure 1.

The complete set of possible confounders is presented in Table S1.

The GEP use across different regions in The Netherlands is illustrated separately for MammaPrint (Figure S1) and Oncotype DX (Figure S1) for patients who met GEP criteria. From 2011 to 2021, MammaPrint was the most used GEP test in all regions. However, in 2022 and 2023, this dynamic shifted, with Oncotype DX becoming the most prevalent in all regions, while the utilisation of MammaPrint declined. Furthermore, in 2024, the proportion of patients who received a MammaPrint increased, whilst the proportion of patients who received an Oncotype DX decreased in certain regions.

Moreover, regional differences in the use of GEPs were observed. Since 2015, the Southwest region has consistently exhibited the highest utilisation of Oncotype DX in comparison to other regions, a trend that has persisted in subsequent years. For MammaPrint, the highest use was seen in the Northwest region.

### 3.2 GEP Use and Chemotherapy

**FIGURE 4:**
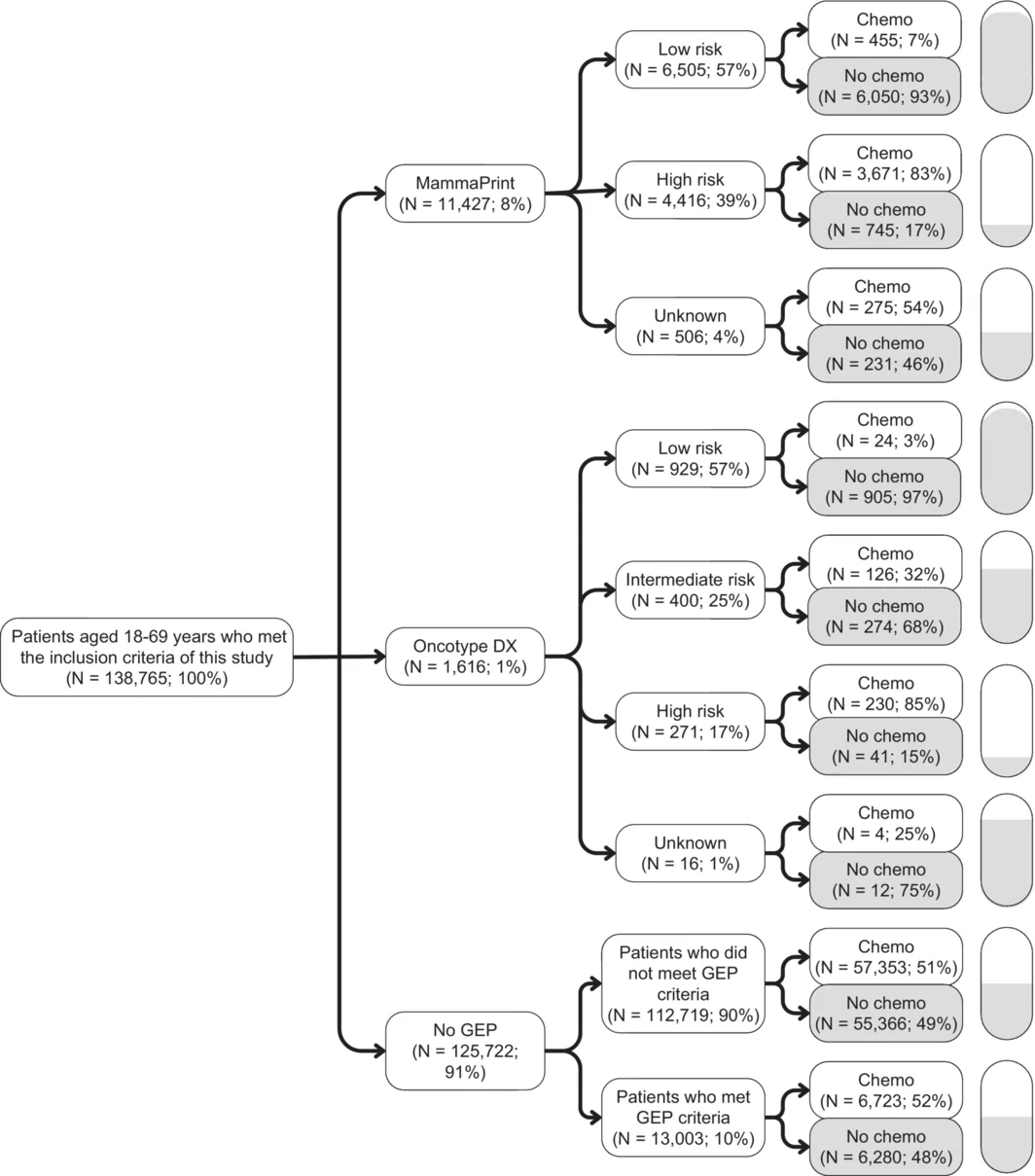
Number of patients grouped according to gene expression profile (GEP) use, GEP result, and chemotherapy use. Eligibility for GEP criteria was based on: Women, older than 50 years, HR+, HER2‐ and either N0 status, grade 1 tumour between 3.1 and 5.0 cm or grade 2 between 2.1–5.0 cm or a grade 3 between 1.1 and 2.0 cm, or N1 status and 1–3 axillary lymph node metastases, a grade 1 tumour between 2.1 and5.0 cm or a grade 2 tumour ≤ 5.0 cm. Hereby, changes in criteria for GEP as well as reimbursement over time were taken into account (Figure 1). The year 2024 comprised solely the months January to June.

Of the total 138,765 included patients, 8% received a MammaPrint, 1% received an Oncotype DX and 91% did not receive a GEP (Figure 4). More precisely, of the 11,427 patients who received a MammaPrint, 57% was considered low risk of whom 93% did not receive chemotherapy, and 39% was considered high risk of whom 17% did not receive chemotherapy. Furthermore, of the 1616 patients who received an Oncotype DX, 57% was considered low risk of whom 97% did not receive chemotherapy, and 17% was considered high risk of whom 15% did not receive chemotherapy. Lastly, of the 125,722 patients who did not receive a GEP, 13,003 met GEP criteria, of whom 52% received chemotherapy. Of.

For patients who did not receive a GEP, those who did not receive chemotherapy were older (median age 60 years), had more often stage 1 (82%), tumour grade 1 (42%) or grade 2 (49%), no lymph node involvement (87%) or a tumour size ≤ 2.0 cm (84%) compared with patients who did receive chemotherapy (Table 1). For patients who received a MammaPrint, those who did not receive chemotherapy had more often stage 1 (52%) or tumour grade 1 (19%) or grade 2 (73%) compared with patients who did receive chemotherapy. For patients who received an Oncotype DX, those who did not receive chemotherapy were older (median age 59 years) and had more often tumour grade 1 (12%) or grade 2 (73%).

## 4 Discussion

The objective of this study was to investigate (1) the utilisation of GEP in the context of reimbursement policies, for both patients with and without an indication for a GEP and (2) GEP use in relation to chemotherapy administration, both among women diagnosed with primary invasive breast cancer without distant metastasis who underwent treatment with curative intent between January 2011 and June 2024 in the Netherlands.

The percentage of patients receiving a GEP increased by 16% after the availability of retrospective reimbursement, and 12% more after publicly known reimbursement, but still approximately only one‐third of the eligible patients received a GEP, despite the evidence that GEP has been proven to be cost‐effective [7, 8, 9, 10]. After reimbursement of GEPs was available, one would expect an increase in GEP use. This increase however was not seen. Although 100% GEP use in patients who met GEP criteria is not expected, for example due to the fact that no chemotherapy is administered due to patient preference and therefore a GEP does not help in shared decision making, this probably does not account for the 63% of patients who do not receive a GEP despite meeting GEP criteria and being eligible for reimbursement. Several reasons may have contributed to the lower‐than‐expected use of GEP. These reasons need to be investigated, and should refer to guideline instructions, other sources used for shared decision making (such as the Predict Tool [22]), and the role of regional policies. Only afterwards can be determined whether the decision not to use GEP was justified. However, this was beyond the scope of the present study. [Correction added on 16 June 2026, after first online publication: In the first sentence of this paragraph, the reported increase in the percentage of patients receiving a GEP has been corrected from “6%” to “12%” in this version.].

Of all 121,240 patients who did not meet GEP criteria and were ineligible for reimbursement, still 7% received a GEP, which was most likely due to the MINDACT study. The MINDACT study demonstrated the effectiveness of MammaPrint in clinical practice and included patients aged 18–70 years and examined T‐status and N‐status, irrespective of subtype, in circumstances where no reimbursement was provided [6]. Thus, women aged 18–50 years were eligible for a GEP in the MINDACT study, but are no longer eligible for a GEP nowadays. Consequently, it is anticipated that a significant proportion of patients categorised as ‘did not meet GEP criteria’ in this present study will have received a MammaPrint after inclusion in the MINDACT study. In addition, results of this study showed that proportion of patients aged ≤ 50 was significantly higher for patients who received a MammaPrint compared to patients who received an Oncotype DX, likely also attributable to the inclusion requirements of the MINDACT study.

Findings of the trends in GEP use over time showed that first of all, between 2012 and 2014, there was an increase in the proportion of patients who received a MammaPrint. This increase can be attributed to the 2012 Dutch breast cancer guideline recommending GEP for patients with ER+ breast cancer tumours [23, 24]. Furthermore, between 2015 and 2016, there was another increase in the proportion of patients who received a MammaPrint. A possible explanation for this can be the publication of the MINDACT study in 2016 [6]. As a result, clinicians and patients could have recognized the positive effects of using MammaPrint in determining treatment, potentially leading to an increase in the use of MammaPrint. This trend was irrespective of whether reimbursement was provided or not. Additionally, from 2021 to 2022, there was a decline in the use of MammaPrint and at the same time an increase in the use of Oncotype DX, as expected due to the reimbursement of Oncotype DX starting in August 2021 while MammaPrint was not yet reimbursed [20]. This reimbursement change most likely influenced clinicians' and patients' preferences, leading them to favour Oncotype DX over MammaPrint. At the same time, this may also be a consequence of the publication of the MINDACT follow‐up study [25], which showed that MammaPrint cannot be used for women under the age of 50. Thereafter, in 2024, the proportion of patients who received a MammaPrint increased again, probably because MammaPrint was reimbursed from October 2023 onwards [11].

In 2020, COVID‐19 affected health care, influencing treatment strategies, early‐stage diagnoses, and the weekly initiation of breast cancer treatments [26, 27]. Despite these disruptions, it seems that the GEP use remained unaffected during the COVID‐19 period. A possible explanation can be that the temporary halt of the screening programme in the Netherlands resulted in a decline of mainly the smaller tumours, which did not influence the patient group eligible for GEP [28, 29, 30, 31].

The findings of this study indicated that the odds of receiving a MammaPrint for patients who were eligible for retrospective or publicly known reimbursement was lower compared with patients who were ineligible for reimbursement. Interestingly, MammaPrint was more frequently used during periods when it was not yet reimbursed. Three factors may explain these results. First, the publication of the MINDACT study in 2016 [6] likely increased clinical confidence in the test's utility, encouraging its use regardless of reimbursement status. Second, there was access to MammaPrint through alternative funding arrangements with support of health insurance companies and hospitals. While reimbursement was not installed from the base‐benefit insurance package on a national level, several insurance companies reimbursed the test from alternative funding because of its clinical utility. Lastly, the reimbursement of Oncotype DX in August 2021—while MammaPrint was still not reimbursed—may have shifted test preference, leading to a decline in MammaPrint use. Collectively, these factors may have contributed to the result that patients eligible for retrospective or publicly known reimbursement were less likely to receive a MammaPrint test compared with those who were ineligible for reimbursement.

Moreover, for patients who received a GEP, results of this study showed that chemotherapy was administered to a lower proportion of patients who had a low‐risk result for MammaPrint (93% no chemotherapy) or Oncotype DX (97% no chemotherapy) compared to those who had a high risk result for MammaPrint (17% no chemotherapy) or for Oncotype DX (15% no chemotherapy), as well as compared with patients who received no GEP (49% no chemotherapy). Therefore, low risk GEP result resulted in a reduction in chemotherapy use. This is in line with a Canadian [7], an Italian [32] and a Dutch [33] study, which demonstrated that using GEP when uncertainty arises regarding chemotherapy's benefits, low risk resulted in a reduction in chemotherapy use.

However, in 6% of the low risk GEP result cases, chemotherapy was still administrated. In such cases, it could be argued that either the GEP was not necessary, or the chemotherapy was not required. A more in‐depth investigation into the motivations behind the decision to give chemotherapy despite a low risk GEP result would be interesting but was out of the scope for this study.

Additionally, of 13,003 patients who met GEP criteria but did not receive a GEP, 52% received chemotherapy. Although it should be noted that not all patients were eligible for reimbursement or patients did not opt for chemotherapy at all, which is most likely the reason why some of these patients did not receive a GEP despite meeting the GEP criteria, it is conceivable that some of these patients could have been spared chemotherapy if they had received a GEP.

Lastly, for patients who received a GEP, results of this study showed that chemotherapy was administered to 83% of the patients who had a high‐risk result for MammaPrint, and to 85% of the patients who had a high‐risk result for Oncotype DX. A recent American study demonstrated a significant chemotherapy benefit in patients with a high‐risk MammaPrint result, and therefore supports the use of MammaPrint as a predictive and prognostic tool for assessing the likelihood of chemotherapy benefit in HR+/HER2‐ early‐stage breast cancer [34]. The advantage of MammaPrint therefore appears to be increasing, with its scope of application expanding.

### 4.1 Strengths and Limitations

By using population‐based data from the NCR, the findings were representative of the general Dutch breast cancer population. The NCR database has an opt‐out procedure for patients, but few patients are removed from the registry, making the risk of selection bias very small.

According to Dutch treatment guidelines, patients aged ≥ 70 years are generally considered ineligible for chemotherapy unless they have no comorbidities and maintain a good performance status [14]. Because information on comorbidities and performance status is not available in the NCR for breast cancer patients, this study could not identify which older patients might still have been eligible for chemotherapy. Therefore, all patients aged ≥ 70 years were excluded. This may have resulted in an underrepresentation of fitter older patients who, in clinical practice, could have been considered for chemotherapy and thus for GEP testing. As a result, our findings mainly reflect GEP use and subsequent chemotherapy use in patients aged < 70 years, limiting insight into the older eligible population. Since GEP testing is reimbursed for all patients aged 50 years and older, future studies should include older patients to better understand how GEP testing informs chemotherapy decision‐making across all eligible age groups who may benefit from it.

There are various possible interpretations of the Oncotype DX results. For example, besides the cutoffs used in this present study, there is also the Sparano outcome measure [35]. However, the NCR registered the cutoffs used in this study, rather than the exact outcome measure. Therefore, it may be a limitation that this is at the expense of comparability with other studies that use Sparano's cutoffs. However, this does not affect the results of this study.

This study highlights an important societal aspect: the implementation of GEPs in routine clinical practice needs to be actively guided to ensure that they are used effectively and uniformly, allowing more patients to benefit from personalized treatment recommendations. Future research should therefore investigate how different implementation strategies, such as standardized clinical pathways and monitoring of uptake, can facilitate the appropriate use of GEP testing in daily practice. Evaluating these approaches in real‐world settings would provide valuable insights into how to optimize implementation and reduce unwarranted variation in GEP use across hospitals.

## 5 Conclusion

Availability of reimbursement for GEP in the Netherlands was found to be somewhat efficacious for the implementation of GEPs. However, approximately only one‐third of the patients who met GEP criteria and were eligible for publicly known reimbursement received a GEP. For patients who received a GEP, results showed that the proportion of patients who had a low risk GEP result that did not receive chemotherapy was higher compared to patients who had a high risk GEP result that did not receive chemotherapy. In conclusion, appropriate follow‐up steps and further research seem required to analyse reasons for not ordering a GEP in times of reimbursement, since GEP results helps chemotherapy decision‐making in clinical practice.

## Author Contributions

**Joyce Meijer:** conceptualization, data curation, formal analysis, project administration, software, supervision, visualization, writing – original draft, writing – review and editing. **Nienke Hermanns:** conceptualization, data curation, formal analysis, software, visualization, writing – original draft, writing – review and editing. **Rhodé Bijlsma:** writing – review and editing, validation. **Hedwig M. Blommestein:** writing – review and editing, validation. **Desiree H. J. G. van den Bongard:** writing – review and editing, validation. **Thijs van Dalen:** writing – review and editing, validation. **Paul J. van Diest:** writing – review and editing, validation. **Caroline A. Drukker:** writing – review and editing, validation. **Cristina Guerrero Paez:** writing – review and editing, validation. **Aafke H. Honkoop:** writing – review and editing, validation. **Agnes Jager:** writing – review and editing, validation. **Linetta B. Koppert:** writing – review and editing, validation. **Sabine C. Linn:** writing – review and editing, validation. **Marissa C. van Maaren:** writing – review and editing, validation. **Ester J. Siemerink:** writing – review and editing, validation. **Laura J. van 't:**writing – review and editing, validation. **José H. Volders:** writing – review and editing, validation. **Marie Jeanne Vrancken Peeters:** writing – review and editing, validation. **Sabine Siesling:** conceptualization, project administration, supervision, writing – original draft, writing – review and editing, validation.

## Ethics Statement

This retrospective study was conducted with pseudomised and aggregated data from the Netherlands Cancer. Registry/Netherlands Comprehensive Cancer Organisation. Data release by the Netherlands Comprehensive. Cancer Organisation is subject to strict regulations, with oversight provided by a Privacy Review Board and Scientific Committee [data request study number 24–00492]. According to Dutch legislation, this study does not fall under the scope of the Medical Research Involving Human Subjects Act (WMO) and therefore did not require approval from a medical ethics review committee.

## Conflicts of Interest

H.M.B. reports research grants outside the submitted work (IMI‐PROTECT trial No 101008134, ZonMw research grants, TeamPerMed Horizon WIDERA 2022, Research grant Zorginstituut Nederland, Research grant Medical Delta), a secondment at HollandPTC, serving as member on the Pfizer BV Advisory Board and being a member of the scientific advisory board medication of the National Healthcare Institute. Payments are all made to the institute. S.C.L. reports research funding paid to the institute from Roche/Genentech, AstraZeneca, BMS, Tesaro, Merck, Immunomedics, Europect Pharmaceuticals, Agendia and Novaris, as consulting role for AstraZeneca/Merck and Agendia paid to the institute, and support for unrestricted educational activities paid to the institute, including coverage for travel and accommodation from Daiichi Sankyo. L.V. reports part‐time employee and stockholder Agendia NV. Other authors declare that they have no known competing financial interests or personal relationships that could have appeared to influence the work reported in this paper.

## Supporting information


**Table S1:** The influence of reimbursement on gene expression profile (GEP)‐use of patients aged 18–69 years and diagnosed with primary invasive breast cancer without distant metastasis between January 2011 and June 2024 treated with curative intent in The Netherlands who did meet GEP criteria* (*N* = 17.525).
**Figure S1:** Proportion of patients aged 18–69 years who met GEP criteria and received (A) MammaPrint or (B) Oncotype DX per incidence year of patients diagnosed with primary invasive breast cancer without distant metastasis between January 2011 and June 2024 treated with curative intent in the Netherlands per region (*N* = 17.525).
