# Supplementary material for: Impact of Reimbursement on the Utilisation of Gene Expression Profiles and Chemotherapy Decision‐Making in Dutch Breast Cancer Patients: A Population‐Based Study
Source: Int J Cancer. 2026 Jun 4;159(10):2485–97. doi: 10.1002/ijc.70571 (PMC13595471; doi:10.1002/ijc.70571)
Supplement: Supplementary file 1 [file IJC-159-2485-s001.pdf]

Impact of reimbursement on the utilisation of gene expression profiles and chemotherapy decision-making in Dutch breast cancer patients: a population-based study

Joyce Meijer, Nienke Hermanns, Rhodé Bijlsma, Hedwig M. Blommestein, Desiree H.J.G. van den Bongard, Thijs van Dalen, Paul J. van Diest, Caroline A. Drukker, Cristina Guerrero Paez, Aafke H. Honkoop, Agnes Jager, Linetta B. Koppert, Sabine C. Linn, Marissa C. van Maaren, Ester J. Siemerink, Laura J. van t Vee, José H. Volders, Marie-Jeanne Vrancken Peeters, Sabine Siesling

Table of contents

Table S1. The influence of reimbursement on gene expression profile (GEP)-use of patients aged 18-69 years and diagnosed with primary invasive breast cancer without distant metastasis between January 2011 and June 2024 treated with curative intent in The Netherlands who did meet GEP criteria* (N=17,525).

Figure S1. Proportion of patients aged 18-69 years who met GEP criteria and received (A) MammaPrint or (B) Oncotype DX per incidence year of patients diagnosed with primary invasive breast cancer without distant metastasis between January 2011 and June 2024 treated with curative intent in the Netherlands per region (N=17,525).

Supplementary Table

Table S1. The influence of reimbursement on gene expression profile (GEP)-use of patients aged 18-69 years and diagnosed with primary invasive breast cancer without distant metastasis between January 2011 and June 2024 treated with curative intent in The Netherlands who did meet GEP criteria* (N=17,525).

		**Odds ratio (95% CI)**	***p*-value**
**MammaPrint**			
Reimbursement periods #	Not eligible for reimbursement	1.00 (reference)	*NA*
	Eligible for retrospective reimbursement	0.97 (0.87 – 1.09)	*0.638*
	Eligible for publicly known reimbursement	0.57 (0.51 – 0.65)	*<0.001*
Age at diagnosis (years)	51-59	1.00 (reference)	*NA*
	60-69	0.79 (0.73 – 0.85)	*<0.001*
Tumour size (cm)	≤ 1.0	0.71 (0.59 – 0.87)	*0.001*
	1.1 – 2.0	1.35 (1.20 – 1.51)	*<0.001*
	2.1 – 3.0	1.00 (reference)	*NA*
	3.1 – 5.0	0.59 (0.52 – 0.67)	*<0.001*
Positive lymph nodes	0	1.00 (reference)	*NA*
	1-3	0.55 (0.49 – 0.62)	*<0.001*
Tumour grade	1	1.13 (0.96 – 1.34)	*0.152*
	2	1.00 (reference)	*NA*
	3	0.69 (0.59 – 0.81)	*<0.001*
SEP	Low	0.80 (0.73 – 0.88)	*<0.001*
	Intermediate	0.92 (0.84 – 1.00)	*0.053*
	High	1.00 (reference)	*NA*
	Unknown	0.78 (0.55 – 1.10)	*0.151*
Region in the Netherlands	Central	0.72 (0.62 – 0.83)	*<0.001*
	Northeast	0.77 (0.68 – 0.87)	*<0.001*
	Northwest	1.00 (reference)	*NA*
	East	0.66 (0.59 – 0.74)	*<0.001*
	West	0.53 (0.46 – 0.62)	*<0.001*
	Southeast	0.54 (0.46 – 0.62)	*<0.001*
	Southwest	0.56 (0.50 – 0.63)	*<0.001*
**Oncotype DX**			
Reimbursement periods #	Not eligible for reimbursement	1.00 (reference)	*NA*
	Eligible for retrospective reimbursement	2.72 (2.11 – 3.50)	*<0.001*
	Eligible for publicly known reimbursement	16.90 (14.08 – 20.29)	*<0.001*
Tumour size (cm)	≤ 1.0	0.40 (0.24 – 0.68)	*0.001*
	1.1 – 2.0	0.92 (0.70 – 1.21)	*0.558*
	2.1 – 3.0	1.00 (reference)	*NA*
	3.1 – 5.0	0.87 (0.73 – 1.17)	*0.222*
Positive lymph nodes	0	1.00 (reference)	*NA*
	1-3	0.92 (0.73 – 1.17)	*0.506*
Tumour grade	1	0.72 (0.49 – 1.06)	*0.092*
	2	1.00 (reference)	*NA*
	3	1.14 (0.82 – 1.60)	*0.431*
Region in the Netherlands	Central	1.02 (0.73 – 1.42)	*0.919*
	Northeast	1.14 (0.85 – 1.51)	*0.379*
	Northwest	1.00 (reference)	*NA*
	East	0.94 (0.70 – 1.24)	*0.646*
	West	0.72 (0.49 – 1.04)	*0.081*
	Southeast	1.28 (0.94 – 1.74)	*0.112*
	Southwest	3.48 (2.76 – 4.39)	*<0.001*

CI, confidence interval; GEP, gene expression profile; NA, not applicable; SEP, socioeconomic position.
* Eligibility for GEP criteria was based on: women, older than 50 years, HR+, HER2-, and either N0 status, grade 1 tumour between 3.1-5.0 cm or grade 2 between 2.1-5.0 cm or a grade 3 between 1.1-2.0 cm, or N1 status and 1-3 axillary lymph node metastases, a grade 1 tumour between 2.1-5.0 cm or a grade 2 tumour ≤5.0 cm. Hereby, changes in criteria for GEP as well as reimbursement over time were taken into account.
# The eligibility criteria for reimbursement were defined in the time path shown in Figure 1.
Multivariable analyses for MammaPrint were adjusted for age, tumour size, lymph node involvement, tumour grade, SEP and region in the Netherlands. For Oncotype DX, multivariable analyses were adjusted for tumour size, lymph node involvement, tumour grade, and region in the Netherlands.
The year 2024 comprised solely the months January to June.

Supplementary Figure

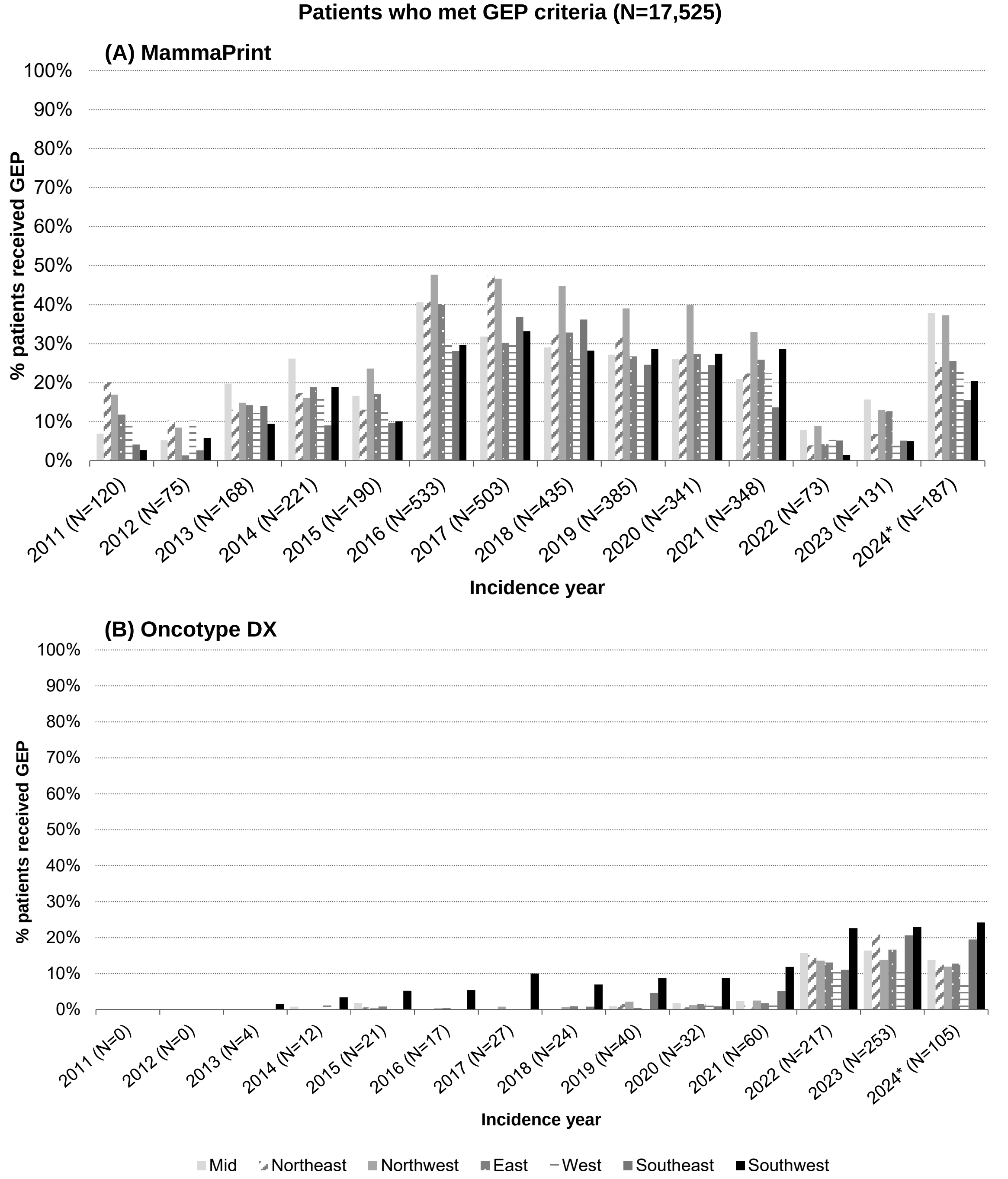

Figure S1. Proportion of patients aged 18-69 years who met GEP criteria and received (A) MammaPrint or (B) Oncotype DX per incidence year of patients diagnosed with primary invasive breast cancer without distant metastasis between January 2011 and June 2024 treated with curative intent in the Netherlands per region (N=17,525).
Eligibility for GEP criteria was based on: women, older than 50 years, HR+, HER2-, and either N0 status, grade 1 tumour between 3.1-5.0 cm or grade 2 between 2.1-5.0 cm or a grade 3 between 1.1-2.0 cm, or N1 status and 1-3 axillary lymph node metastases, a grade 1 tumour between 2.1-5.0 cm or a grade 2 tumour ≤5.0 cm. Hereby, changes in criteria for GEP as well as reimbursement over time were taken into account (Figure 1).
N represents the number of patients who received a GEP.
* The year 2024 comprised solely the months January to June.
